# Clinical utility of the neutrophil elastase inhibitor sivelestat for the treatment of ALI/ARDS patients with COVID-19

**DOI:** 10.1016/j.heliyon.2024.e36337

**Published:** 2024-08-20

**Authors:** Ruiying Wang, Junping Yin, Jian Li, Xueli Bai, Hu Liu, Mengyu Cheng, Lei Wang, Yuan Chen, Shuang Wei, Xiansheng Liu

**Affiliations:** a Department of Pulmonary and Critical Care Medicine, Shanxi Bethune Hospital, Shanxi Academy of Medical Sciences, Tongji Shanxi Hospital, Third Hospital of Shanxi Medical University, Taiyuan, Shanxi, China; b Sino-German Joint Oncological Research Laboratory, Shanxi Bethune Hospital, Shanxi Academy of Medical Sciences, Taiyuan, Shanxi, China; c Institute of Molecular Medicine and Experimental Immunology, University Clinic of Rheinische Friedrich-Wilhelms-University, Bonn, Germany; d Department of Geriatrics, Tongji Hospital, Tongji Medical College, Huazhong University of Science and Technology, 1096 Jiefang Road, Wuhan, Hubei, China; e Department of Pulmonary and Critical Care Medicine,Tongji Hospital, Tongji Medical College, Huazhong University of Science and Technology, Wuhan, Hubei, China

**Keywords:** COVID-19, Sivelestat, Treatment, ARDS, Lung injury

## Abstract

**Background:**

Sivelestat, a neutrophil elastase inhibitor, is postulated to mitigate acute lung injury in patients following emergency surgery. However, its efficacy in patients with acute lung injury (ALI) and acute respiratory distress syndrome (ARDS) induced by coronavirus disease 2019 (COVID-19) remains uncertain. This study aims to evaluate the pulmonary protective effects of sivelestat in COVID-19 patients with ALI/ARDS.

**Methods:**

A retrospective study was conducted involving 2454 COVID-19 patients between October 5, 2022, and February 1, 2023. Of these, 102 patients received sivelestat (0.2 mg/kg/h), while 2352 age- and sex-matched controls were identified. Propensity score matching (PSM) analysis was used to match sivelestat and non-sivelestat subgroups in ratios of 1:1 and 1:3 for sensitivity analysis. The primary outcome was a composite of effective outcomes, including 30-day mortality. Secondary outcomes included changes in partial pressure of arterial oxygen (PaO_2_), the ratio of PaO_2_ to the fraction of inspired oxygen (PaO_2_/FiO_2_), and various cytokine levels. Safety evaluations included assessments of liver function, kidney function, and leukopenia.

**Results:**

In the propensity score-matched analysis, the sivelestat group had a higher proportion of severe/critical patients (87.26 % vs. 51.02 %, *P* < 0.001), more ARDS patients (4.9 % vs. 0.43 %, *P* < 0.001), and more patients with interstitial lung disease (4.9 % vs. 1.49 %, *P* = 0.023), but fewer patients with stroke (17.65 % vs. 19.86 %, *P* < 0.001). Oxygen therapy rates were similar between the groups (79.41 % vs. 80.95 %, *P* = 0.9). The relative risk reduction in 30-day mortality was 88.45 % (95 % confidence interval [CI] 81.23%–93.21 %) for severe/critical COVID-19 patients treated with sivelestat. Sivelestat significantly decreased cytokine levels of interferon alpha (IFNα), interleukin-1 beta (IL-1β), and interleukin-2 (IL-2).In the sivelestat group, the mortality rate was significantly reduced with standard oxygenation and HFNC therapy(*P* < 0.05). The treatment with sivelestat did not increase side effects.

**Conclusion:**

The administration of the neutrophil elastase inhibitor sivelestat may improve clinical outcomes in COVID-19 patients with ALI/ARDS. These findings suggest that sivelestat could be considered an effective treatment option to alleviate pulmonary inflammatory injury caused by severe acute respiratory syndrome coronavirus 2 (SARS-CoV-2).

## 1 Introduction

Acute Respiratory Distress Syndrome (ARDS) is a severe form of acute lung injury (ALI), often characterized by extensive damage to the alveolar epithelium and capillary endothelium, leading to pulmonary edema and the formation of hyaline membranes, leading to impaired gas exchange and accompanied by the release of systemic inflammatory mediators [1]. It is one of the most prevalent conditions in the intensive care unit (ICU) and a leading cause of morbidity and mortality worldwide [2]. Although various causes or risk factors can lead to ALI/ARDS, the most common are pneumonia and non-pulmonary sepsis [3]. The COVID-19 pandemic has exacerbated the incidence of ARDS, with 42 % of COVID-19 patients presenting with pneumonia [4], highlighting the syndrome's challenges, its unacceptably high mortality, and the lack of effective pharmacotherapy.

Sivelestat is a selective, reversible, and competitive neutrophil elastase inhibitor [5,6], demonstrating protective effects in attenuating ALI/ARDS. Studies have shown that sivelestat can lower the production of inflammatory cytokines such as IL-1β, IL-6, and TNF-α [7], and reduce the infiltration and activation of inflammatory cells [8], thereby protecting against post-perfusion-induced lung injury [9]. Furthermore, sivelestat therapy might positively impact the PaO_2_/FiO_2_ ratio, although it appears to have no significant effect on 28–30 day mortality, ventilation days, and ICU stays in ARDS induced by the SARS virus [10]. Recently, some researchers suggested that neutrophil elastase inhibitors might be a potential prophylactic treatment for COVID-19 patients [11], based on evidence from basic research indicating the inhibitory effects of sivelestat on NF-κB signaling [[12], [13], [14]]. However, clinical studies have produced controversial results regarding the efficacy of sivelestat in treating ARDS [15]. Current evidence suggests that sivelestat may offer some benefit in ALI/ARDS treatment, but large-scale clinical trials are needed to explore these potential benefits in specific pathophysiological conditions.

To address this issue, we conducted a retrospective cohort study to evaluate the efficacy and safety of sivelestat in treating COVID-19 patients with ALI/ARDS.

## 2 Methods

### 2.1 Study population

This retrospective cohort study was approved by the ethical committee of Shanxi Bethune Hospital (approval number: 2022-102-01). The inclusion criteria were: (1) patients confirmed with a positive nucleic acid test for SARS-CoV-2 using reverse transcriptase PCR and subsequently admitted to the hospital; (2) chest imaging showing lesions in both lungs. The exclusion criteria were: (1) patients who died, were anticipated to be discharged, transferred to another hospital, or received standard care for ≤48 h after diagnosis; (2) pregnancy or breastfeeding; (3) known allergy.

COVID-19 was clinically categorized into several stages: mild to moderate disease encompassing both non-pneumonia and pneumonia presentations; severe disease characterized by dyspnea, respiratory frequency exceeding 30 breaths per minute, oxygen saturation below 93 %, PaO_2_/FiO_2_ ratio less than 300, and/or lung infiltrates over 50 % within 24–48 h; and critical illness involving respiratory failure, septic shock, and/or multi-organ dysfunction/failure [16,17].

The diagnostic criteria for ARDS are as follows [18]:(1)Acute onset (within 7 days of new or worsening respiratory symptoms).(2) Bilateral radiographic opacities not fully explained by effusion, atelectasis, or masses.(3)Arterial hypoxemia defined by the following thresholds:Mild: 200 < PaO_2_/FiO_2_ ratio ≤300 mm Hg, CPAP/PEEP≥5 cm H_2_O.Moderate: 100 < PaO_2_/FiO_2_ ratio≤200 mm Hg, PEEP≥5 cm H_2_O.Severe: PaO_2_/FiO_2_ ratio≤100 mm Hg, PEEP≥5 cm H_2_O.(4)Identified risk factor for ARDS (if no clear risk factor, exclude heart failure as a cause). (5)Condition not exclusively due to cardiac causes.

### 2.2 Procedures

According to the application instructions for sivelestat, dissolve the drug with a daily dose (4.8 mg/kg) in 0.9 % sodium chloride, then use a 50 ml syringe to draw and hold the sivelestat solution. Afterwards, dilute this solution with 250–500 ml normal saline to a total volume of 500 ml, ready for use. Set the dosing rate to 2 ml/h with the intravenous injection micropump for a 24-h constant rate of injection. The daily dosage can be divided into 3 parts and administered via continuous intravenous infusion, with the longest course of drug administration being 14 days. The last follow-up date was 3^th^March 2023.

Patients are stratified and assigned to different respiratory support modalities based on their oxygenation index and the severity of their condition. The gradation of respiratory support includes:(1)Standard oxygenation (nasal cannula/mask: Initial treatment for patients with mild to moderate hypoxemia.(2)High-flow nasal cannula (HFNC,up to 60 L/min)): Upgraded from standard oxygen therapy if patients fail to maintain adequate oxygenation levels.(3)Non-Invasive Mechanical Ventilation (NIV): Utilized when HFNC is insufficient to alleviate respiratory distress.(4)Invasive Mechanical Ventilation with Lung-Protective Strategy: Implemented as a last resort if the patient's condition continues to worsen despite NIV.Patients are grouped according to the most advanced level of respiratory support they receive.

### 2.3 Biological sample collection

Biological samples were collected at two specific times: before any treatment was initiated upon patient admission and 14 days after the treatment.

### 2.4 Data collection

Survival time was calculated from the date of hospital admission to death from any cause. The primary outcome was to evaluate efficacy by examining the 30-day mortality rate. The secondary outcomes included observing changes in PaO_2_, PaO_2_/FiO_2_ ratios, and various cytokine levels. Safety evaluations were performed, focusing on liver function injury, kidney function injury, and leukopenia.

### 2.5 Statistical analysis

All statistical analyses were performed using the latest versions of Stata, Easy-R, and R (version 4.0). GraphPad (https://www.graphpad.com/scientific-software/prism/) was used for drawing purposes. Continuous variables were presented as mean ± standard deviation (SD) or median with interquartile ranges (IQRs), while categorical variables were presented as proportions (%). Continuous data were analyzed using t-tests or rank-sum tests, and qualitative data were analyzed using chi-square tests and/or Fisher's exact chi-square tests. Multivariate linear regression, logistic regression, and Cox proportional hazards models were applied to analyze risk ratios and hazard risk ratios.

To mitigate potential biases that could affect our findings, we used propensity score matching (PSM) to adjust the study cohort. This methodology allowed for a comparison between patients who received sivelestat and those in the control group with a similar risk profile. PSM analysis was performed to match sivelestat and non-sivelestat groups in ratios of 1:1 and 1:3 for sensitivity analysis. A P-value of less than 0.05 was considered statistically significant.

## 3 Results

### 3.1 Clinical characteristics of COVID-19 patients

**Fig. 1:**
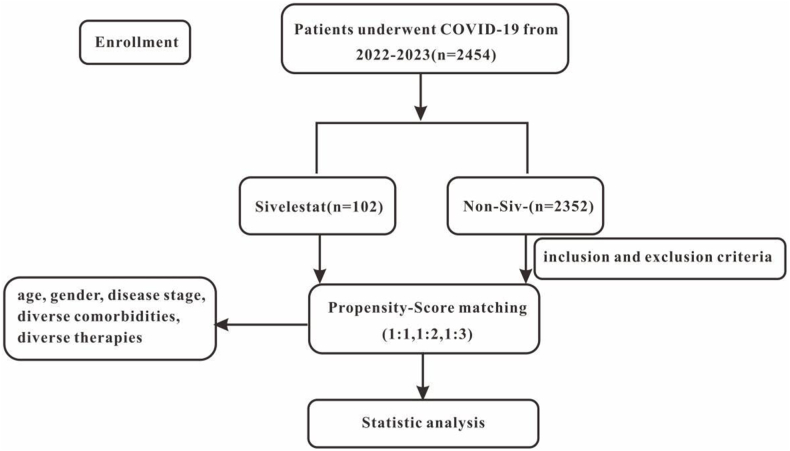
Workflow of study design.

Between 5th October 2022, and 1st February 2023, a total of 2731 COVID-19 patients were screened at Shanxi Bethune Hospital. Ultimately, 2454 hospitalized COVID-19 patients were included in the analysis (Fig. 1). Nearly everyone has been vaccinated. Specifically, 80.9 % of patients have completed the full course of COVID-19 vaccination, while 19.1 % have received only one dose.Among them, 102 patients received sivelestat (sivelestat group), while 2352 patients did not receive this drug (non-sivelestat group). All patients completed the follow-up.

In this cohort, the median age was 69.0 years (IQR 57.0–81.0). Of the total, 1323 patients (53.91 %) were male, 510 (20.78 %) were current smokers, and 1289 (52.53 %) experienced a severe/critical disease course.(Table 1). Common comorbidities associated with increased risk of progression to severe/critical COVID-19 at baseline included interstitial lung disease (ILD) in 40 patients (1.63 %), chronic obstructive pulmonary disease (COPD) in 78 patients (3.18 %), hypertension in 823 patients (33.54 %), coronary heart disease (CHD) in 140 patients (5.70 %), stroke in 485 patients (19.76 %), diabetes mellitus (DM) in 667 patients (27.18 %), malignant tumors in 323 patients (13.16 %), hematologic disorders in 490 patients (19.97 %), renal dysfunction in 231 patients (9.41 %), and liver dysfunction in 262 patients (10.68 %).(Table 2, Table 3).

**Table 1:** Characteristics in pre-propensity matching.

		Total (N = 2454)	Non-Siv (N = 2352)	Sivelestat (N = 102)	Pvalue
Age	mean ± sd	65,34 ± 19,60	64,99 ± 19,78	73,27 ± 12,66	5.13E-09
	min	0	0	28	
	max	102	102	96	
	median	69,00 (57,00, 81,00)	69,00 (57,00, 80,00)	75,00 (63,00, 84,00)	
Sex	Male	1323 (53,91 %)	1261 (53,61 %)	62 (60,78 %)	0.186556783968456
	Female	1131 (46,09 %)	1091 (46,39 %)	40 (39,22 %)	
Smoking	No	1915 (78,04 %)	1830 (77,81 %)	85 (83,33 %)	0.285388933310671
	Yes	510 (20,78 %)	493 (20,96 %)	17 (16,67 %)	
Diseases severity	Moderate	1165 (47,47 %)	1152 (48,98 %)	13 (12,75 %)	1.94E-13
	Sever	1167 (47,56 %)	1092 (46,43 %)	75 (73,53 %)	
	More than sever	122 (4,97 %)	108 (4,59 %)	14 (13,73 %)	
ILD	No	2414 (98,37 %)	2317 (98,51 %)	97 (95,10 %)	0.0234333718633592
	Yes	40 (1,63 %)	35 (1,49 %)	5 (4,90 %)	
COPD	No	2375 (96,78 %)	2277 (96,81 %)	98 (96,08 %)	0.889730561616863
	Yes	78 (3,18 %)	74 (3,15 %)	4 (3,92 %)	
ARDS	No	2439 (99,39 %)	2342 (99,57 %)	97 (95,10 %)	4.90E-07
	Yes	15 (0,61 %)	10 (0,43 %)	5 (4,90 %)	
Hypertension	No	1622 (66,10 %)	1554 (66,07 %)	68 (66,67 %)	0.940889570629115
	Yes	823 (33,54 %)	789 (33,55 %)	34 (33,33 %)	
CHD	No	2305 (93,93 %)	2211 (94,01 %)	94 (92,16 %)	0.734197566933726
	Yes	140 (5,70 %)	132 (5,61 %)	8 (7,84 %)	
Stroke	No	1968 (80,20 %)	1885 (80,14 %)	83 (81,37 %)	8.64E-06
	Yes	485 (19,76 %)	467 (19,86 %)	18 (17,65 %)	
DM	No	1787 (72,82 %)	1712 (72,79 %)	75 (73,53 %)	0.95943807531091
	Yes	667 (27,18 %)	640 (27,21 %)	27 (26,47 %)	
Malignant tumors	No	2131 (86,84 %)	2036 (86,56 %)	95 (93,14 %)	0.0762888677637222
	Yes	323 (13,16 %)	316 (13,44 %)	7 (6,86 %)	
Hematologic disorders	No	1963 (79,99 %)	1880 (79,93 %)	83 (81,37 %)	0.921076008801307
	Yes	490 (19,97 %)	471 (20,03 %)	19 (18,63 %)	
Renal dysfunction	No	2221 (90,51 %)	2127 (90,43 %)	94 (92,16 %)	0.819751541039117
	Yes	231 (9,41 %)	223 (9,48 %)	8 (7,84 %)	
Liver dysfunction	No	2190 (89,24 %)	2106 (89,54 %)	84 (82,35 %)	0.0640516683547692
	Yes	262 (10,68 %)	244 (10,37 %)	18 (17,65 %)

**Table 2:** Characteristics in after-propensity matching(Ratio 1:1).

		Total (N = 204)	Non-Siv (N = 102)	Sivelestat (N = 102)	pvalue
Age	mean ± sd	73,08 ± 12,42	72,88 ± 12,23	73,27 ± 12,66	0.8222
	min	28	28	28	
	max	96	96	96	
	median	75,00 (63,00, 84,00)	75,00 (63,00, 83,00)	75,00 (63,00, 84,00)	
Sex	Male	126 (61,76 %)	64 (62,75 %)	62 (60,78 %)	0.885442848627866
	Female	78 (38,24 %)	38 (37,25 %)	40 (39,22 %)	
Smoking	No	159 (77,94 %)	74 (72,55 %)	85 (83,33 %)	0.0913084289068644
	Yes	45 (22,06 %)	28 (27,45 %)	17 (16,67 %)	
Diseases severity	Moderate	28 (13,73 %)	15 (14,71 %)	13 (12,75 %)	0.8621
	Sever	150 (73,53 %)	75 (73,53 %)	75 (73,53 %)	
	More than sever	26 (12,75 %)	12 (11,76 %)	14 (13,73 %)	
ILD	No	198 (97,06 %)	101 (99,02 %)	97 (95,10 %)	0.213807729542997
	Yes	6 (2,94 %)	1 (0,98 %)	5 (4,90 %)	
COPD	No	188 (92,16 %)	90 (88,24 %)	98 (96,08 %)	0.0683113132443061
	Yes	16 (7,84 %)	12 (11,76 %)	4 (3,92 %)	
ARDS	No	199 (97,55 %)	102 (100,00 %)	97 (95,10 %)	0.0701117534576819
	Yes	5 (2,45 %)	0 (0,00 %)	5 (4,90 %)	
Hypertension	No	125 (61,27 %)	57 (55,88 %)	68 (66,67 %)	0.196906626251612
	Yes	78 (38,24 %)	44 (43,14 %)	34 (33,33 %)	
CHD	No	186 (91,18 %)	92 (90,20 %)	94 (92,16 %)	0.582652425626489
	Yes	17 (8,33 %)	9 (8,82 %)	8 (7,84 %)	
Stroke	No	164 (80,39 %)	81 (79,41 %)	83 (81,37 %)	0.533882374191182
	Yes	39 (19,12 %)	21 (20,59 %)	18 (17,65 %)	
DM	No	145 (71,08 %)	70 (68,63 %)	75 (73,53 %)	0.536784864740017
	Yes	59 (28,92 %)	32 (31,37 %)	27 (26,47 %)	
Malignant tumors	No	185 (90,69 %)	90 (88,24 %)	95 (93,14 %)	0.335229125597609
	Yes	19 (9,31 %)	12 (11,76 %)	7 (6,86 %)	
Hematologic disorders	No	160 (78,43 %)	77 (75,49 %)	83 (81,37 %)	0.405271658738583
	Yes	43 (21,08 %)	24 (23,53 %)	19 (18,63 %)	
Renal dysfunction	No	180 (88,24 %)	86 (84,31 %)	94 (92,16 %)	0.128222894999772
	Yes	24 (11,76 %)	16 (15,69 %)	8 (7,84 %)	
Liver dysfunction	No	172 (84,31 %)	88 (86,27 %)	84 (82,35 %)	0.563561202900117
	Yes	32 (15,69 %)	14 (13,73 %)	18 (17,65 %)

**Table 3:** Characteristics in after-propensity matching(Ratio 1:3).

		Total (N = 408)	Non-Siv (N = 306)	Sivelestat (N = 102)	pvalue
Age	mean ± sd	72,88 ± 12,71	72,75 ± 12,75	73,27 ± 12,66	0.7204
	min	28	28	28	
	max	96	96	96	
	median	75,00 (63,00, 83,25)	75,50 (63,00, 83,00)	75,00 (63,00, 84,00)	
Sex	Male	260 (63,73 %)	198 (64,71 %)	62 (60,78 %)	0.552177684126288
	Female	148 (36,27 %)	108 (35,29 %)	40 (39,22 %)	
Smoking	No	317 (77,70 %)	232 (75,82 %)	85 (83,33 %)	0.215304054357068
	Yes	88 (21,57 %)	71 (23,20 %)	17 (16,67 %)	
Diseases severity	Moderate	59 (14,46 %)	46 (15,03 %)	13 (12,75 %)	0.8006
	Sever	298 (73,04 %)	223 (72,88 %)	75 (73,53 %)	
	More than sever	51 (12,50 %)	37 (12,09 %)	14 (13,73 %)	
ILD	No	399 (97,79 %)	302 (98,69 %)	97 (95,10 %)	0.0798639029693502
	Yes	9 (2,21 %)	4 (1,31 %)	5 (4,90 %)	
COPD	No	380 (93,14 %)	282 (92,16 %)	98 (96,08 %)	0.25823482340595
	Yes	28 (6,86 %)	24 (7,84 %)	4 (3,92 %)	
ARDS	No	401 (98,28 %)	304 (99,35 %)	97 (95,10 %)	0.0154669277030268
	Yes	7 (1,72 %)	2 (0,65 %)	5 (4,90 %)	
Hypertension	No	247 (60,54 %)	179 (58,50 %)	68 (66,67 %)	0.30471021704024
	Yes	160 (39,22 %)	126 (41,18 %)	34 (33,33 %)	
CHD	No	378 (92,65 %)	284 (92,81 %)	94 (92,16 %)	0.802394840028027
	Yes	29 (7,11 %)	21 (6,86 %)	8 (7,84 %)	
Stroke	No	321 (78,68 %)	238 (77,78 %)	83 (81,37 %)	0.143320848249861
	Yes	86 (21,08 %)	68 (22,22 %)	18 (17,65 %)	
DM	No	295 (72,30 %)	220 (71,90 %)	75 (73,53 %)	0.84803999836173
	Yes	113 (27,70 %)	86 (28,10 %)	27 (26,47 %)	
Malignant tumors	No	360 (88,24 %)	265 (86,60 %)	95 (93,14 %)	0.110294255244555
	Yes	48 (11,76 %)	41 (13,40 %)	7 (6,86 %)	
Hematologic disorders	No	321 (78,68 %)	238 (77,78 %)	83 (81,37 %)	0.654888934725812
	Yes	86 (21,08 %)	67 (21,90 %)	19 (18,63 %)	
Renal dysfunction	No	364 (89,22 %)	270 (88,24 %)	94 (92,16 %)	0.495794882734941
	Yes	43 (10,54 %)	35 (11,44 %)	8 (7,84 %)	
Liver dysfunction	No	352 (86,27 %)	268 (87,58 %)	84 (82,35 %)	0.244881188235494
	Yes	56 (13,73 %)	38 (12,42 %)	18 (17,65 %)	

### 3.2 Laboratory results and treatment of COVID-19 patients

Table 4 summarizes the results of neutrophil, lymphocyte, and D-dimer levels, which are key indicators in this study. Key treatments administered to the cohort during the study period included glucocorticoid therapy in 63.65 % (1562 patients), antiviral therapy in 53.10 % (1303 patients), and oxygen therapy in 80.89 % (1985 patients) as detailed in Table 5, Table 6.

**Table 4:** All Results of laboratory tests and therapeutic regimens.

		Total (N = 2454)	Non-Siv(N = 2352)	Sivelestat (N = 102)	Pvalue
Neutrophil	mean ± sd	5,14 ± 4,20	5,06 ± 4,11	6,85 ± 5,71	9.62E-177
	min	0	0	0.67	
	max	78.66	78.66	38.85	
	median (IQR)	4,18 (2,83, 6,34)	4,16 (2,80, 6,27)	5,53 (3,49, 7,91)	
Lymphocyte	mean ± sd	1,11 ± 1,89	1,13 ± 1,93	0,83 ± 0,74	5.67E-53
	min	0	0	0.04	
	max	63.32	63.32	5.92	
	median (IQR)	0,90 (0,55, 1,38)	0,92 (0,56, 1,39)	0,62 (0,37, 1,07)	
Oxygen_saturation	mean ± sd	92,77 ± 7,44	92,77 ± 7,49	92,72 ± 6,25	4.74E-34
	min	26.3	26.3	55.4	
	max	101.7	101.7	99	
	median (IQR)	94,50 (91,50, 96,60)	94,50 (91,50, 96,62)	94,30 (91,03, 96,60)	
D_Dimer	mean ± sd	802,05 ± 2,361,24	800,44 ± 2,393,51	841,78 ± 1,347,19	1.32E-164
	min	9	9	77	
	max	65353	65353	7860	
	median (IQR)	303,00 (151,00, 691,00)	297,50 (147,75, 691,00)	412,00 (253,75, 710,25)	
Anti_virus	No	1098 (44,74 %)	1070 (45,49 %)	28 (27,45 %)	0.00159861653134677
	Yes	1303 (53,10 %)	1232 (52,38 %)	71 (69,61 %)	
Glucocorticoids	No	892 (36,35 %)	887 (37,71 %)	5 (4,90 %)	3.15E-11
	Yes	1562 (63,65 %)	1465 (62,29 %)	97 (95,10 %)	
Oxygen_therapy	No	468 (19,07 %)	447 (19,01 %)	21 (20,59 %)	0.904597473111583
	Yes	1985 (80,89 %)	1904 (80,95 %)	81 (79,41 %)

**Table 5:** Laboratory tests and therapeutic regimens(PSM matched ratio 1:1).

		Total (N = 204)	Non-Siv (N = 102)	Sivelestat (N = 102)	pvalue
Neutrophil	mean ± sd	6,24 ± 4,90	5,63 ± 3,87	6,85 ± 5,71	2.87E-59
	min	0.01	0.01	0.67	
	max	38.85	28.02	38.85	
	median (IQR)	5,09 (3,24, 7,51)	4,67 (3,12, 6,60)	5,53 (3,49, 7,91)	
Lymphocyte	mean ± sd	1,16 ± 4,43	1,49 ± 6,22	0,83 ± 0,74	6.65E-38
	min	0.04	0.07	0.04	
	max	63.32	63.32	5.92	
	median (IQR)	0,67 (0,40, 1,13)	0,71 (0,42, 1,15)	0,62 (0,37, 1,07)	
Oxygen_saturation	mean ± sd	179; 92,19 ± 6,91	97; 91,74 ± 7,43	92,72 ± 6,25	1.79E-36
	min	47.1	47.1	55.4	
	max	99.2	99.2	99	
	median (IQR)	179; 93,50 (90,50, 95,80)	97; 93,20 (90,30, 95,50)	94,30 (91,03, 96,60)	
D_Dimer	mean ± sd	183; 922,52 ± 2,302,99	97; 994,11 ± 2,904,66	841,78 ± 1,347,19	1.74E-52
	min	45	45	77	
	max	25300	25300	7860	
	median (IQR)	183; 378,00 (211,50, 709,00)	97; 335,00 (159,00, 694,00)	412,00 (253,75, 710,25)	
Anti_virus	No	60 (29,41\%)	32 (31,37\%)	28 (27,45 %)	0.597090685861612
	Yes	136 (66,67\%)	65 (63,73\%)	71 (69,61 %)	
Glucocorticoids	No	8 (3,92\%)	3 (2,94\%)	5 (4,90 %)	0.718326262969495
	Yes	196 (96,08\%)	99 (97,06\%)	97 (95,10 %)	
Oxygen_therapy	No	28 (13,73\%)	7 (6,86\%)	21 (20,59 %)	0.00816947251470263
	Yes	176 (86,27\%)	95 (93,14\%)	81 (79,41 %)

**Table 6:** Laboratory tests and therapeutic regimens(PSM matched ratio 1:3).

		Total (N = 204)	Non-Siv (N = 102)	Sivelestat (N = 102)	Pvalue
Neutrophil	mean ± sd	6,24 ± 4,90	5,63 ± 3,87	6,85 ± 5,71	2.87E-59
	min	0.01	0.01	0.67	
	max	38.85	28.02	38.85	
	median (IQR)	5,09 (3,24, 7,51)	4,67 (3,12, 6,60)	5,53 (3,49, 7,91)	
Lymphocyte	mean ± sd	1,16 ± 4,43	1,49 ± 6,22	0,83 ± 0,74	6.65E-38
	min	0.04	0.07	0.04	
	max	63.32	63.32	5.92	
	median (IQR)	0,67 (0,40, 1,13)	0,71 (0,42, 1,15)	0,62 (0,37, 1,07)	
Oxygen_saturation	mean ± sd	179; 92,19 ± 6,91	97; 91,74 ± 7,43	92,72 ± 6,25	1.79E-36
	min	47.1	47.1	55.4	
	max	99.2	99.2	99	
	median (IQR)	179; 93,50 (90,50, 95,80)	97; 93,20 (90,30, 95,50)	94,30 (91,03, 96,60)	
D_Dimer	mean ± sd	183; 922,52 ± 2,302,99	97; 994,11 ± 2,904,66	841,78 ± 1,347,19	1.74E-52
	min	45	45	77	
	max	25300	25300	7860	
	median (IQR)	183; 378,00 (211,50, 709,00)	97; 335,00 (159,00, 694,00)	412,00 (253,75, 710,25)	
Anti_virus	No	60 (29,41\%)	32 (31,37\%)	28 (27,45 %)	0.597090685861612
	Yes	136 (66,67\%)	65 (63,73\%)	71 (69,61 %)	
Glucocorticoids	No	8 (3,92\%)	3 (2,94\%)	5 (4,90 %)	0.718326262969495
	Yes	196 (96,08\%)	99 (97,06\%)	97 (95,10 %)	
Oxygen_therapy	No	28 (13,73\%)	7 (6,86\%)	21 (20,59 %)	0.00816947251470263
	Yes	176 (86,27\%)	95 (93,14\%)	81 (79,41 %)	

### 3.3 Comparison between sivelestat group and control group

Compared to the non-sivelestat subgroup, the sivelestat subgroup was significantly older (median 75.0 vs. 69.0, *P* < 0.001) and had a higher proportion of severe/critical COVID-19 patients (87.26 % vs. 51.02 %, *P* < 0.001). Additionally, the sivelestat subgroup had a higher prevalence of ARDS patients (4.9 % vs. 0.43 %, *P* < 0.001) and interstitial lung disease (ILD) patients (4.9 % vs. 1.49 %, *P* < 0.05), but a lower prevalence of stroke (17.65 % vs. 19.86 %, *P* < 0.001). The sivelestat group also received more glucocorticoid therapy (95.1 % vs. 62.29 %, *P* < 0.001) and anti-virus therapy (69.61 % vs. 52.38 %, *P* = 0.0016), while oxygen therapy usage was similar between the two groups (79.41 % vs. 80.95 %, *P* = 0.9). Neutrophil counts and D-dimer levels were higher in the sivelestat group compared to the non-sivelestat group. Conversely, lymphocyte counts were lower in the sivelestat group. Clinical outcomes appeared comparable between both subgroups (Table 1, Table 2, Table 3, Table 4).

### 3.4 The primary outcome-Sivelestat reduce the overall mortality

**Fig. 2:**
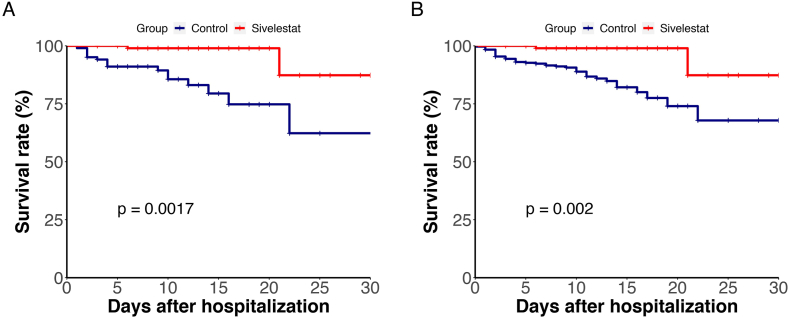
Kaplan-Meier Plot of overall survival of sivelestat treatment. (A, B) PSM matched ratio 1:1 and 1:3 between sivelestat and n**on-sivelestat** subgroups.

To facilitate a comprehensive comparison, propensity score matching (PSM) analysis was employed to pair both subgroups 1:1 based on age, gender, disease severity, various comorbidities, diverse therapeutic interventions, and other relevant variables. This approach led to the selection of 204 patients (102 pairs) for evaluating the efficacy of sivelestat treatment. The majority of patients in both matched subgroups presented with severe/critical disease. Significantly fewer patients in the matched sivelestat subgroup experienced mortality by the study endpoint (6 deaths) compared to the matched non-sivelestat subgroup (17 deaths; 5.88 % vs. 16.67 %, *P* = 0.0017). Kaplan-Meier analysis demonstrated a reduction in the hazard ratio (HR) to 0.25 (95 % CI 0.095–0.63, *P* = 0.0017) with sivelestat treatment (Fig. 2A). Regarding the recovery rate, no discernible differences were observed between the matched subgroups.For a more detailed analysis, PSM was also conducted using a 1:3 matched ratio, revealing 42 deaths in the matched non-sivelestat subgroup. The corresponding HR was estimated at 0.28 (95 % CI 0.12–0.66, *P* = 0.002) (Fig. 2B). In summary, sivelestat treatment resulted in an 88.45 % relative risk reduction in mortality (95 % CI 81.23 %–93.21 %) among severe/critical COVID-19 patients.

**Fig. 3:**
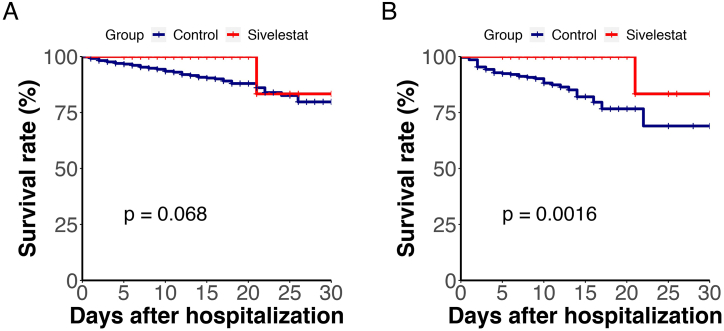
Kaplan-Meier Plot of overall survival of patients receiving oxygen therapy. (A, B) PSM matched ratio 1:1 and 1:3 between sivelestat and n**on-sivelestat** subgroups.

Applying the same statistical methods, after excluding patients who did not undergo oxygen therapy, the overall survival rate did not show significant differences (Fig. 3A: HR 0.362, 95 % CI 0.115–1.139, *P* = 0.068). However, propensity score matching (PSM) with a 1:3 ratio analysis demonstrated that the survival rate in the sivelestat group remained superior to that in the control group (Fig. 3B: HR 0.182, 95 % CI 0.056–0.595, *P* = 0.0015).

### 3.5 Sivelestat reduce the mortality of ARDS patients

**Fig. 4:**
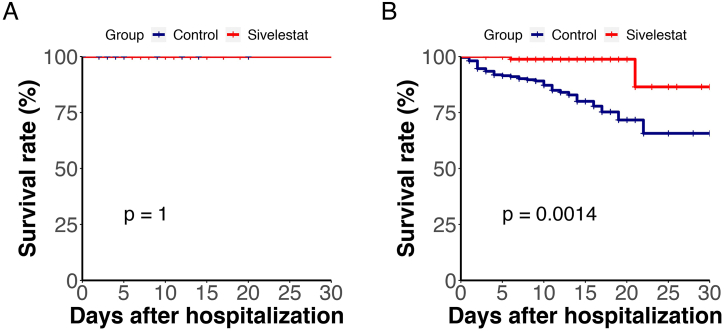
Kaplan-Meier Plot of survival of patients with different severity levels. (A, B) A illustrates the comparison of survival rates among patients with moderate COVID-19; Figure B presents the comparison of survival rates among patients with severe and critical COVID-19.

In this study, all ARDS patients were included among the severe and critical COVID-19 cases. We conducted a survival rate comparison among moderate, severe, and critical COVID-19 patients and found no statistically significant difference in survival rates for moderate COVID-19 patients after using Sivelestat(Fig. 4A) However, for severe and critical patients, the survival rate in the Sivelestat group was significantly higher than in the control group(Fig. 4B, *P* = 0.0014**)**.Therefore, we speculate that the use of Sivelestat is effective for ARDS.

### 3.6 Optimized survival with high-flow/standard oxygenation in sivelestat group

**Fig. 5:**
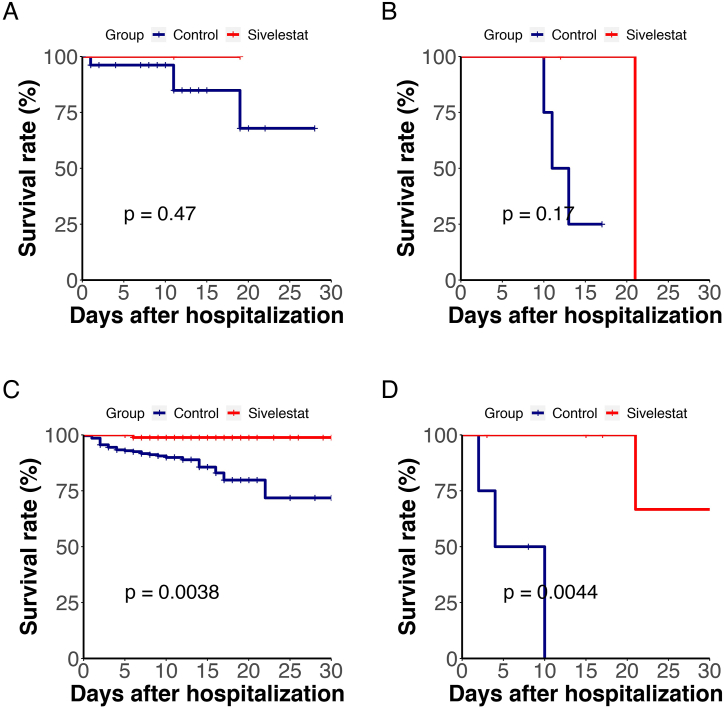
Kaplan-Meier Plot of survival of patients with different Different oxygen treatments.Figure A represents patients treated with invasive ventilators; Figure B with non-invasive ventilators; Figure C with nasal cannula/mask oxygenation; Figure D with high-flow oxygen therapy. (**Note:** Duration in Fig A and B is reduced due to classification based on the highest level of respiratory support.).

In this study, the methods of oxygen therapy primarily included the use of invasive ventilators, non-invasive ventilators, standard oxygenation(nasal cannula/mask), and High-flow nasal cannula (HFNC). We conducted an analysis of the different oxygen therapy modalities beweent sivelestat group and control group.There was no statistically significant difference in survival rates using invasive ventilation and non-invasive ventilators(Fig. 5A/B).In the Sivelestat group, the mortality rate was significantly reduced with standard oxygenation (Fig. 5C, *P* = 0.0038) and HFNC therapy(Fig. 5D, *P* = 0.0044**)**

### 3.7 Sivelestat improve PaO_2_/FiO_2_ and alleviate the IFNα、IL-1β and IL-2 levels

**Fig. 6:**
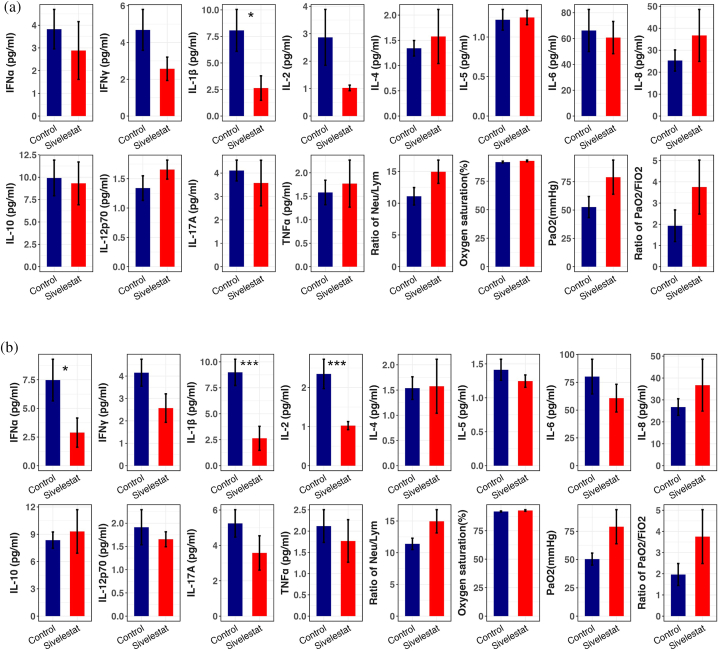
Barplot of all samples. Clinical and laboratory parameters'comparison between Treatment and Control group. Here, one can observe that some cytokines such as IFNα、IL-1β and IL-2 decreased significantly in sivelestat group than that in Control group, whereas Others cytokine did not differ significantly between both groups.(*P < 0.05,**P < 0.01,***P < 0.001).Fig. 6A. Barplots of matched samples by ratio 1:1 Fig. 6B. Barplots of matched samples by ratio 1:3.

Compared to the non-sivelestat group, the sivelestat group showed significant reductions in cytokine levels of IFNα, IL-1β, and IL-2 (p < 0.05), indicating a potential modulation of inflammatory responses. Additionally, sivelestat treatment was associated with improved levels of PaO_2_/FiO_2_, suggesting enhanced oxygenation efficiency in these patients.However, no statistically significant differences were observed between the two groups in cytokines such as IL-8, IL-10, IL-17A, IL-4, IL-5, IL-6, IL-12, and TNF-a (Fig. 6/6A/6B). These findings suggest that while sivelestat may effectively target specific cytokines and improve oxygenation parameters, its impact on other inflammatory markers remains inconclusive in this study cohort.

### 3.8 Factors affecting mortality in COVID-19 patients: multivariate analysis

Multivariate Cox regression analysis highlighted several critical factors associated with mortality in the cohort of COVID-19 patients. Advanced age, elevated D-dimer levels (≥1500 ng/ml), reduced lymphocyte counts (≤0.8*10^9^/L), increased neutrophil counts (>7*10^9^/L), severe/critical disease status, and the presence of ARDS emerged as significant predictors of mortality. Importantly, the absence of sivelestat treatment was also identified as a contributing factor to mortality, underscoring its potential protective role in COVID-19 patients with ARDS(Table 7, Table 8).

**Table 7:** Univariant. COX analysis.

	Total				Ratio 1:1				Ratio 1:3			
	HR	HR.confint.lower	HR.confint.upper	P.value	HR	HR.confint.lower	HR.confint.upper	P.value	HR	HR.confint.lower	HR.confint.upper	P.value
Sivelestat	0.59	0.26	1.3	0.21	0.25	0.095	0.63	0.0035	0.28	0.12	0.66	0.0037
Diseases_severity	22	9.7	50	1.3E-13	3.7	0.48	29	0.21	7.4	1	54	0.049
Sex	1.6	1.1	2.2	0.01	1.3	0.51	3.1	0.61	0.99	0.54	1.8	0.98
Age_group	2.1	1.5	2.9	0.0000056	1.6	0.67	3.7	0.3	1.7	0.96	3	0.071
Smoking	1.3	0.89	1.8	0.19	1.8	0.72	4.5	0.21	1.5	0.8	2.8	0.2
ILD	3.1	1.5	6.3	0.002	0.00000011	0	inf	1	1.3	0.17	9.3	0.81
COPD	1.5	0.72	3.3	0.26	0.65	0.087	4.9	0.67	0.55	0.13	2.3	0.41
ARDS	5.7	2.7	12	0.0000081	1.8	0.23	13	0.58	1.3	0.31	5.6	0.71
Hypertension	1.1	0.77	1.5	0.67	1.1	0.44	2.6	0.87	0.95	0.52	1.7	0.87
Stroke	1.4	1	2	0.051	2.6	1.1	6.4	0.038	2.2	1.2	4	0.013
DM	0.92	0.65	1.3	0.67	0.83	0.32	2.1	0.71	1.1	0.6	2.1	0.73
Malignant_tumors	0.95	0.62	1.5	0.83	0.49	0.11	2.2	0.35	1.2	0.57	2.7	0.58
Hematologic_disorders	1.2	0.87	1.8	0.24	0.75	0.27	2.1	0.58	0.88	0.44	1.7	0.71
Renal_dysfunction	1.9	1.3	2.9	0.0022	1.6	0.53	4.7	0.42	1.6	0.73	3.4	0.25
Liver_dysfunction	1.4	0.92	2.1	0.12	1.1	0.39	3	0.87	1.2	0.61	2.5	0.55
CHD	1.2	0.64	2.2	0.59	0.23	0.029	1.8	0.17	0.83	0.29	2.4	0.74
Anti_virus	1.2	0.89	1.7	0.21	1.2	0.49	2.9	0.69	1.1	0.55	2	0.88
Glucocorticoids	1.7	1.2	2.5	0.006	1	0.22	5	0.96	0.78	0.22	2.7	0.7
Oxygen_therapy	1.5	0.94	2.4	0.087	1.6	0.45	5.5	0.47	1	0.45	2.3	0.98
D_Dimer_group	3.1	2.1	4.5	0.0000000023	1.4	0.39	4.8	0.62	2.4	1.2	4.9	0.012
Lym_group	0.3	0.21	0.43	3.7E-11	0.58	0.24	1.4	0.23	0.57	0.31	1.1	0.079
Neu_group	3.3	2.4	4.5	5.6E-13	1.9	0.83	4.5	0.13	2	1.1	3.5	0.022

**Table 8:**
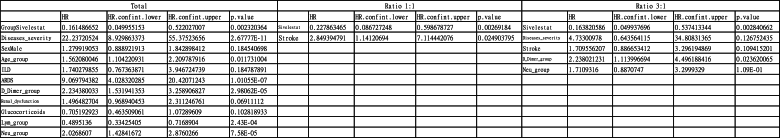
Multivariant. COX analysis.

### 3.9 No increased side effects in the sivelestat group

Sivelestat treatment may potentially lead to certain adverse effects, primarily manifesting as liver function abnormalities indicated by elevated levels of AST, ALT, ALP, and Bilirubin. It can also sporadically cause kidney-related concerns such as increased urea and creatinine levels or the presence of protein in urine. Specifically, levels exceeding the upper limit of normal(AST>40 IU/L, ALT> 40 IU/L, ALP >135 U/L,bilirubin >21 μmol/L,Urea>7.1 mmol/L, Creatinine>96 mmol/L)are considered abnormal.Hematological issues, including decreased blood cell counts with white blood cells below 4 × 10^9^/L and platelets below 100 × 10^9^/L,have also been observed. Symptomatic therapies are available for managing these side effects, and discontinuation of the medication is considered if necessary.

The incidence of adverse effects observed during and after the treatment course was comparable between the sivelestat and non-sivelestat subgroups (Table 9). Among the sivelestat subgroup, the most frequently reported events included liver injury (33.3 %, compared to 30.9 % in the control subgroup, *P* = 0.587), kidney injury (27.5 % vs. 25.6 %, *P* = 0.729), thrombocytopenia (20.6 % vs. 14.8 %, *P* = 0.120), and leukopenia (10.8 % vs. 10.4 %, *P* = 0.868). There was one reported death in the sivelestat subgroup and 31 deaths in the entire control subgroup (0.70 % vs. 1.34 %, *P* = 0.516). No significant differences were observed in adverse events affecting other organs between the two subgroups.The adverse effects/events reported in this cohort were generally not considered specifically related to sivelestat treatment by the site investigators. The severity of these adverse effects/events was not distinctly differentiated during the study, as most were self-resolving and required specific treatment in only a few cases. Furthermore, no adverse effect/event led to discontinuation of treatment in this study.

**Table 9:** Adverse events in the current cohort.

Treatment-emergent adverse effect	Control (n = 2352)	Sivletestat treatment (n = 102)	P-value
	N(%)	N(%)	
Liver-injury	727 (30.9)	34 (33.3)	0.587
Kidney-injury	603 (25.6)	28 (27.5)	0.729
Urine protein	143 (6.1)	6 (5.9)	1
Leukocytopenia	244 (10.4)	11 (10.8)	0.868
Thrombopenia	349 (14.8)	21 (20.6)	0.12

### 3.10 Multiple studies of ALI presented sivelestat can improve clicinal outcome

In our study, only 102 patients received sivelestat treatment, reflecting its limited usage. Given the current lack of clarity regarding the clinical efficacy of sivelestat in ALI and ARDS caused by COVID-19, we conducted a literature review to explore its effectiveness.A systematic search using keywords ‘sivelestat’, ‘acute lung injury','ARDS' and ‘randomized controlled trial (RCT)' on PubMed identified 10 eligible RCTs reporting on sivelestat.Among these, 7 RCTs were centered on post-surgery settings, where sivelestat was linked with improved PaO_2_/FiO_2_ ratios at 24 h, reduced duration of systemic inflammatory response syndrome (SIRS), and lower concentrations of polymorphonuclear elastase. However, findings regarding IL-6/IL-8 levels in these studies were inconsistent, and there was no consistent association observed with in-hospital mortality.Additionally, 2 RCTs explored sivelestat in cases of ALI associated with SIRS, demonstrating improved weaning rates from mechanical ventilation in the ICU. These collective findings underscore sivelestat's potential to enhance clinical outcomes. Nevertheless, further research is needed due to its limited application and the small sample sizes in reported studies.Detailed characteristics of these RCTs are summarized in **Supplementary Table。**

## 4 Discussion

Results from this retrospective cohort study demonstrated the efficacy of sivelestat administration in hospitalized patients with pneumonia induced by COVID-19. The clinical outcomes showed significant efficacy of sivelestat treatment, with an 88.45 % reduction in the risk of death from any cause, particularly among patients who progressed to ARDS. This efficacy was assessed through multiple PSM analyses with ratios of 1:1 and 1:3, which effectively minimized the confounding effects of clinical variables including age, gender, comorbidities, disease stage, combined therapies, and other factors. The majority of patients in the PSM groups exhibited severe/critical COVID-19 conditions. Furthermore, sivelestat treatment was observed to reduce the release of inflammatory cytokines and improve oxygenation status in these patients.

ARDS caused by COVID-19 represents a severe inflammatory response, significantly increasing both morbidity and mortality [3,4]. Upon viral invasion, neutrophils typically mount aggressive responses, migrating rapidly to affected tissues, particularly the lungs, where they release neutrophil extracellular traps and generate reactive oxygen species (ROS) through oxidative bursts [19]. Neutrophil activation plays a pivotal role in this process.In light of these mechanisms, Sahebnasagh and colleagues [20] have suggested that sivelestat, a neutrophil elastase inhibitor, holds promise as a therapeutic option for managing ALI/ARDS in COVID-19. Sivelestat is known to effectively mitigate lung tissue damage and suppress the production of inflammatory mediators in ARDS. Studies have demonstrated its protective effects against lung injury in animal models [21]. However, clinical research on sivelestat's application in COVID-19-induced ARDS remains limited.Among those hospitalized with COVID-19, 15–30 % will progress to COVID-19-associated ARDS [22].Severe COVID-19 pneumonia, as characterized by the National Institutes of Health (NIH),1 exhibits substantial overlap with the clinical manifestations of “typical” ARDS [23].Our study identified that all patients meeting ARDS criteria were exclusively present in the severe and critical categories of COVID-19. Notably, the survival rate among severe and critical patients treated with Sivelestat was significantly higher than that of the control group. These results indicate that Sivelestat administration significantly improves survival in ALI/ARDS patients following COVID-19, particularly when treatment is initiated early. The findings position Sivelestat as a potential therapeutic intervention for protecting lung function and treating inflammatory lung injury in COVID-19, warranting further clinical investigation.

Activated neutrophils can migrate back into the bloodstream via neutrophil reverse transendothelial migration [24], potentially contributing to multiorgan failure by causing distant organ damage. Moreover, activated neutrophils release cytokines, elastase, and superoxide [25,26], which increase vascular permeability, leading to interstitial lung edema [27,28].In our study, patients treated with sivelestat showed reduced levels of cytokines such as IFNα, IL-1β, and IL-2, consistent with previous findings [7,8,25]. The sivelestat group also exhibited lower plasma IL-6 levels, suggesting an anti-inflammatory effect. However, the impact on IL-6 levels was not consistently significant across 1:1 and 1:3 propensity score matches, possibly due to the time-dependent nature of IL-6 response to treatment. The relationship between IL-6 and neutrophil elastase inhibitors remains debated [29].Furthermore, our findings indicated an improvement in the PaO_2_/FiO_2_ ratio in sivelestat-treated patients, indicating enhanced oxygenation and improved prognosis. Interestingly, a separate study involving 167 septic patients with ARDS demonstrated that sivelestat improved lung injury scores, PaO_2_/FiO_2_ ratios, DIC scores, ICU length of stay, and survival rates [30]. Similarly, studies on ALI/ARDS post-surgery reported favorable outcomes with sivelestat, including reduced pneumonia incidence, shorter mechanical ventilation duration, decreased bilateral pulmonary infiltrates, improved oxygenation index, and lower IL-6 levels [31,32].While supplemental oxygen therapy is crucial for ARDS, especially in COVID-19-related cases where HFNC is widely favored clinically, our study similarly found higher survival rates with HFNC use [33]. However, due to limited ventilator availability during the pandemic and the retrospective nature of our study, details regarding invasive and noninvasive respiratory support are unclear. Consequently, caution is needed when interpreting our findings, which show no statistically significant differences in respiratory support types among these patients. Severe respiratory failure in COVID-19 patients can be heterogeneous, with some patients developing bacterial pneumonia and others thrombotic coagulopathy. SARS-CoV-2 mutations also lead to different pulmonary symptoms.These factors complicate predicting sivelestat's treatment effects from broad-syndrome trials [34].Despite these promising observations, robust evidence supporting the widespread use of neutrophil elastase inhibitors in COVID-19-induced ARDS remains insufficient. While our study suggests potential benefits of sivelestat in improving outcomes for COVID-19-related ARDS patients, rigorous randomized prospective trials are essential to validate these findings and establish clear treatment guidelines in the future.

During the study period, no serious adverse effects or events necessitating discontinuation of treatment were reported. The most commonly observed adverse effects were organ damage, although these were deemed by the site investigators to be unrelated to sivelestat treatment. Notably, patients with COVID-19-induced ALI/ARDS frequently experience compromised immune function, potentially leading to secondary infections [35] or disturbances in venous circulation [36]. Therefore, our findings suggest that sivelestat may offer a safe treatment option for ARDS induced by COVID-19.These observations are particularly significant given the context of ARDS in COVID-19 patients, where the immune system's response is often dysregulated. The ability of sivelestat to manage inflammatory responses without significant adverse effects is promising. Future studies should continue to monitor for any potential adverse effects comprehensively and explore sivelestat's safety profile in larger cohorts to validate its suitability as a therapeutic intervention for COVID-19-related ARDS.

## 5 Study limitation

The present study has several limitations that warrant consideration. Firstly, it is based on data from a single center, which may restrict the generalizability of our findings due to the relatively modest sample size, despite employing propensity score matching to mitigate selection bias. Secondly, a majority of severe/critical COVID-19 cases in our study were associated with the emerging Omicron variant of SARS-CoV-2. Whether sivelestat's efficacy in treating ALI/ARDS extends to other SARS-CoV-2 variants or different viruses like Influenza remains uncertain.Thirdly, owing to the retrospective design, specific details such as the treatment duration and timelines for administering sivelestat and Oxygen therapy following ARDS diagnosis are unclear and should be interpreted cautiously. Fourthly, the geographic homogeneity of our patient cohort suggests caution in extrapolating these findings globally. Large-scale international studies are needed to validate our study outcomes. Lastly, our study did not delve into the detailed molecular mechanisms underlying sivelestat's inhibition of virus intrusion or replication. Further research is necessary to elucidate these mechanisms comprehensively.

## 6 Conclusion

ALI/ARDS induced by various viruses presents a persistent clinical challenge. The administration of neutrophil elastase inhibitors, such as sivelestat, represents a potential avenue to enhance clinical outcomes in COVID-19 patients with ALI/ARDS. Our findings suggest that sivelestat could be a viable treatment option for mitigating pulmonary inflammatory injury caused by SARS-CoV-2. Further research is needed to confirm these findings and explore sivelestat's broader applications and underlying mechanisms in managing viral-induced respiratory distress.

## Data availability

Data included in article.

## Ethics statement

This study was approved by the ethical committee of Shanxi Bethune Hospital (number: 2022-102-01).The ethics committee had provide a waiver for informed consent due to the retrospective study.

## Funding

This project was supported by 2023 COVID-19 Emergency Project of Shanxi province Health Commission (No.2023XG001 to Xiansheng Liu,No.2023XG005 to Shuang Wei).Four“batches”innovation project of invigorating medical through science and technology of shanxi province China (2023XM003 to Xiansheng Liu)

## CRediT authorship contribution statement

**Ruiying Wang:** Writing – original draft, Formal analysis. **Junping Yin:** Formal analysis, Data curation. **Jian Li:** Investigation, Formal analysis. **Xueli Bai:** Investigation. **Hu Liu:** Investigation. **Mengyu Cheng:** Methodology, Data curation. **Lei Wang:** Investigation. **Yuan Chen:** Visualization, Supervision. **Shuang Wei:** Writing – review & editing, Funding acquisition. **Xiansheng Liu:** Project administration, Funding acquisition.

## Declaration of competing interest

All authors declare no conflict of interest related to the present study.

## Abbreviations in alphabetical order

ALI: Acute Lung Injury
ALP: Alkaline Phosphatase
ALT: Alanine Aminotrans ferase
ARDS: Acute Respiratory Distress Syndrome
AST: Aspartate Aminotransferase
CHD: Coronary Heart Disease
COPD: Chronic Obstructive Pulmonary Disease
COVID-19: Coronavirus Disease 2019
DIC: Disseminated Intravascular Coagulation
DM: Diabetes Mellitus
FiO_2_: Fraction of Inspired Oxygen
IFNα: Interferon Alpha
IL-1β: Interleukin 1 Beta
IL-2: Interleukin 2
IL-4: Interleukin 4
IL-5: Interleukin 5
IL-6: Interleukin 6
IL-8: Interleukin 8
IL-10: Interleukin 10
IL-12: Interleukin 12
IL-17A: Interleukin 17A
NF-κB: Nuclear Factor Kappa B
PaO_2_: Partial Pressure of Oxygen
PaO_2_/FiO_2_: Ratio of Partial Pressure of Oxygen to Fraction of Inspired Oxygen
PEEP: Positive End-Expiratory Pressure
ROS: Reactive Oxygen Species
SARS-CoV-2: Severe Acute Respiratory Syndrome Coronavirus 2
SIRS: Systemic Inflammatory Response Syndrome
