# Supplementary material for: Clinical utility of the neutrophil elastase inhibitor sivelestat for the treatment of ALI/ARDS patients with COVID-19
Source: Heliyon. 2024 Aug 20;10(17):e36337. doi: 10.1016/j.heliyon.2024.e36337 (PMC11408777; doi:10.1016/j.heliyon.2024.e36337)
Supplement: Multimedia component 1 [file mmc1.docx]

**Table 1**: Effectiveness evaluation of sivelestat in prevention of acute lung injury in randomized controlled trials.

| Study | NCT number | Drug | Condition | Clinical endpoint | intervention | N | Estimation Parameter | Estimation  Value | *P* value |
| --- | --- | --- | --- | --- | --- | --- | --- | --- | --- |
| Abe 2009 |  | Sivelestat | on-pump coronary  artery bypass | Postoperative hospital stay (days) | PBO | 10 | Mean difference | 25.6 ± 9.1 | 0.04 |
|  |  |  |  |  | 0.3 mg/kg/h IV | 10 |  | 19.0 ± 3.4 |  |
|  |  |  |  | Alveolar–arte  rial oxygen gradient (mmHg) at 2 h after arrival to the intensive care unit | PBO | 10 | Mean difference | 279.9 ± 113.3 | 0.131 |
|  |  |  |  |  | 0.3 mg/kg/h IV | 10 |  | 303.6 ± 86.8 |  |
|  |  |  |  | Intrapulmonary shunt (%) at 2 h after arrival to the intensive care unit | PBO | 10 | Mean difference | 18.4 ±4.6 | 0.259 |
|  |  |  |  |  | 0.3 mg/kg/h IV | 10 |  | 19.3 ±5.9 |  |
|  |  |  |  | Dynamic lung compliance (ml/cm H_2_O) at 2 h after arrival to the intensive care unit | PBO | 10 | Mean difference | 32.6 ±6.2 | 0.316 |
|  |  |  |  |  | 0.3 mg/kg/h IV | 10 |  | 35.9 ±7.5 |  |
| Morimoto 2011 |  | sivelestat  sodium hydrate | thoracic aortic surgery | PaO2/FiO2 ratios at 72 hours after surgery | PBO | 12 | Mean difference | 191 ± 49 | > 0.05 |
|  |  |  |  |  | 0.2 mg/kg/h IV | 10 |  | 145 ± 55 |  |
|  |  |  |  | The change in the P/F ratio at 24 hours after surgery versus the baseline ratio value (%) | PBO | 12 | Mean difference | 18 ± 10 | 0.02 |
|  |  |  |  |  | 0.2 mg/kg/h IV | 10 |  | 59 ± 10 |  |
|  |  |  |  | in-hospital mortality (%) | PBO | 12 | Proportion | 17 | > 0.05 |
|  |  |  |  |  | 0.2 mg/kg/h IV | 10 |  | 10 |  |
|  |  |  |  | the duration of  mechanical ventilation (hours) | PBO | 12 | Mean difference | 224 ± 247 | 0.722 |
|  |  |  |  |  | 0.2 mg/kg/h IV | 10 |  | 174 ± 141 |  |
|  |  |  |  | the length of  stay in ICU (days) | PBO | 12 | Mean difference | 39 ± 41 | 0.054 |
|  |  |  |  |  | 0.2 mg/kg/h IV | 10 |  | 18 ± 17 |  |
|  |  |  |  | the length of hospital stay (days) | PBO | 12 | Mean difference | 90 ± 65 | 0.124 |
|  |  |  |  |  | 0.2 mg/kg/h IV | 10 |  | 56 ± 42 |  |
| Fujii 2010 |  | Sivelestat | aortic valve replacement | Changes in concentrations of PMN elastase | PBO | 6 | Mean difference |  | 0.001 |
|  |  |  |  |  | 0.2 mg/kg/h IV | 6 |  |  |  |
|  |  |  |  | Changes in levels of interleukin-8 | PBO | 6 | Mean difference |  | 0.022 |
|  |  |  |  |  | 0.2 mg/kg/h IV | 6 |  |  |  |
|  |  |  |  | Changes in levels of interleukin-6 | PBO | 6 | Mean difference |  | 0.042 |
|  |  |  |  |  | 0.2 mg/kg/h IV | 6 |  |  |  |
|  |  |  |  | Changes in PaO  yFIO throughout the clinical course | PBO | 6 | Mean difference |  | 0.054 |
|  |  |  |  |  | 0.2 mg/kg/h IV | 6 |  |  |  |
| Yamaguchi 2011 |  | Sivelestat | Esophagectomy | Changes in the serum levels of IL-6 | PBO | 12 | Mean difference |  |  |
|  |  |  |  |  | 0.2 mg/kg/h IV | 12 |  |  |  |
|  |  |  |  | Changes in the serum levels of IL-8 | PBO | 12 | Mean difference |  |  |
|  |  |  |  |  | 0.2 mg/kg/h IV | 12 |  |  |  |
|  |  |  |  | Changes in the IL-6 levels in the epithelial lining fluid | PBO | 12 | Mean difference |  |  |
|  |  |  |  |  | 0.2 mg/kg/h IV | 12 |  |  |  |
|  |  |  |  | Changes in the IL-8 levels in the epithelial lining fluid | PBO | 12 | Mean difference |  |  |
|  |  |  |  |  | 0.2 mg/kg/h IV | 12 |  |  |  |
|  |  |  |  | Changes in the levels of the PaO2/FIO2 ratio | PBO | 12 |  |  |  |
|  |  |  |  |  | 0.2 mg/kg/h IV | 12 |  |  |  |
|  |  |  |  | Changes in the levels of respiratory index | PBO | 12 |  |  |  |
|  |  |  |  |  | 0.2 mg/kg/h IV | 12 |  |  |  |
| Kadoi 2004 |  | ONO-5046 |  | Ventilation days (days) | PBO | 12 | Mean difference | 23.5 ± 5.0 | 0.08 |
|  |  |  |  |  | 0.2 mg/kg/h IV | 12 |  | 19.2 ± 6.8 |  |
|  |  |  |  | ICU stay (days) | PBO | 12 | Mean difference | 29.9 ±24.7 | 0.085 |
|  |  |  |  |  | 0.2 mg/kg/h IV | 12 |  | 24.9 ±14.7 |  |
|  |  |  |  | Mortality (%) | PBO | 12 | Proportion | 25 | > 0.05 |
|  |  |  |  |  | 0.2 mg/kg/h IV | 12 |  | 25 |  |
|  |  |  |  | changes in Pao2/  Fio2 ratio | PBO | 12 | Mean difference | 207 ± 69 | > 0.05 |
|  |  |  |  |  | 0.2 mg/kg/h IV | 12 |  | 241 ± 73 |  |
|  |  |  |  | changes in neutrophil elastase activity | PBO | 12 | Mean difference | 202 ± 153 | < 0.05 |
|  |  |  |  |  | 0.2 mg/kg/h IV | 12 |  | 82 ± 70 |  |
|  |  |  |  | changes in IL-6 levels | PBO | 12 | Mean difference | 193 ± 76 | > 0.05 |
|  |  |  |  |  | 0.2 mg/kg/h IV | 12 |  | 111 ± 124 |  |
| Kawahara 2010 |  |  | video-assisted thoracoscopic surgery | ICU stay  (hours) | PBO | 10 | Mean difference | 74.5 (39.0–109.0) | 0.481 |
|  |  |  |  |  | 300 mg/day IV | 10 | Mean difference | 64.0 (39.0–109.0) |  |
|  |  |  |  | Hospital stay  (days) | PBO | 10 | Mean difference | 31 (18–81) | 0.853 |
|  |  |  |  |  | 300 mg/day IV | 10 |  | 32 (19–46) |  |
|  |  |  |  | SIRS (hours) | PBO | 10 | Mean difference | 49 (15–60) | 0.009 |
|  |  |  |  |  | 300 mg/day IV | 10 |  | 17 (9–36) |  |
|  |  |  |  | The PaO2/  FiO2 ratio at 24 hours after surgery | PBO | 10 | Mean difference | 322.5 (243.5–380.0) | 0.040 |
|  |  |  |  |  | 300 mg/day IV | 10 |  | 372.0 (284.0–475.0) |  |
| Makino 2011 | NCT01170845 | sivelestat  sodium hydrate | video-assisted  thoracoscopic oesophagect my for cancer | The ratio of the arterial partial pressure of oxygen to the fractional concentration of inspired oxygen | PBO | 15 |  |  | < 0.050 |
|  |  |  |  |  | 4·8 mg/kg daily for 7 days | 16 |  |  |  |
|  |  |  |  | Length of ICU stay (days) | PBO | 15 | Mean difference | 8·1 (5·7, 10·6) | 0·048 |
|  |  |  |  |  | 4·8 mg/kg daily for 7 days | 16 |  | 5·4 (4·0, 6·9) |  |
|  |  |  |  | hospital stay (days) | PBO | 15 | Mean difference | 45·9 (31·2, 60·7) | 0·077 |
|  |  |  |  |  | 4·8 mg/kg daily for 7 days | 16 |  | 32·6 (26·4, 38·8) |  |
|  |  |  |  | Duration of SIRS (days) | PBO | 15 | Mean difference | 5·5 (4·3, 6·7) | 0·001 |
|  |  |  |  |  | 4·8 mg/kg daily for 7 days | 16 |  | 2·7 (2·0, 3·4) |  |
|  |  |  |  | Duration of mechanical ventilation (h) | PBO | 15 | Mean difference | 170·9 (85·6, 256·1) | 0·046 |
|  |  |  |  |  | 4·8 mg/kg daily for 7 days | 16 |  | 85·6 (57·0, 114·2) |  |
| Ryugo 2006 |  | Sivelestat Sodium | cardiovascular surgery | PaO2/FiO2 | PBO | 7 | Mean difference | 287 ± 39 | - |
|  |  |  |  |  | 0.2 mg/kg/h IV | 7 |  | 218 ± 33 |  |
|  |  |  |  | PMN elastase (pg/ml) | PBO | 7 | Mean difference | 4 799 ± 2839 | - |
|  |  |  |  |  | 0.2 mg/kg/h IV | 7 | Mean difference | 1 162 ± 645 |  |
|  |  |  |  | Respiratory index | PBO | 7 | Mean difference | 1.68 ± 0.48 | - |
|  |  |  |  |  | 0.2 mg/kg/h IV | 7 | Mean difference | 2.34 ± 0.47 |  |
|  |  |  |  | IL-8 (pg/ml) | PBO | 7 | Mean difference | 90.9 ± 27.1 | - |
|  |  |  |  |  | 0.2 mg/kg/h IV | 7 | Mean difference | 44.5 ± 10.6 |  |
|  |  |  |  | WBC counts (/mm3 ) | PBO | 7 | Mean difference | 12 350 ± 1205 | - |
|  |  |  |  |  | 0.2 mg/kg/h IV | 7 | Mean difference | 12 424 ± 567 |  |
|  |  |  |  | Expression of CD18 (%) | PBO | 7 | Mean difference | 94.2 ± 2.2 | - |
|  |  |  |  |  | 0.2 mg/kg/h IV | 7 |  | 95.7 ± 2.14 |  |
| Tamakuma 2004 Study 1 |  | Sivelestat sodium hydrate | ALI associated with SIRS | Change of the pulmonary function normalization rate | PBO | 108 |  |  | 0.0022 |
|  |  |  |  |  | 0.2 mg/kg/h IV | 113 |  |  |  |
|  |  |  |  | The weaning rate from mechanical ventilation | PBO | 108 |  |  | 0.0400 |
|  |  |  |  |  | 0.2 mg/kg/h IV | 113 |  |  |  |
|  |  |  |  | The ICU discharge rate | PBO | 108 |  |  | 0.0495 |
|  |  |  |  |  | 0.2 mg/kg/h IV | 113 |  |  |  |
|  |  |  |  | survival rate | PBO | 108 |  |  | 0.1913 |
|  |  |  |  |  | 0.2 mg/kg/h IV | 113 |  |  |  |
| Tamakuma 2004 Study 2 |  | Sivelestat sodium hydrate | ALI associated with SIRS | Ventilation free days | PBO | 61 | Mean difference | 10.7 ± 10.8 | - |
|  |  |  |  |  | 0.2 mg/kg/h IV | 46 |  | 13.1 ± 10.9 |  |
|  |  |  |  | Relationship between the VFD value the ICU free days and the VFD value | PBO | 61 | correlation coefficient | 0.928 | 0.0001 |
|  |  |  |  |  | 0.2 mg/kg/h IV | 46 |  |  |  |
